# Supplementary material for: Geomagnetic Field (GMF)-Dependent Modulation of Iron-Sulfur Interplay in Arabidopsis thaliana
Source: Int J Mol Sci. 2021 Sep 21;22(18):10166. doi: 10.3390/ijms221810166 (PMC8469209; doi:10.3390/ijms221810166)
Supplement: Supplementary file 1 [file ijms-22-10166-s001.zip › ijms-1386884-supplementary.pdf]

Table S1. Two-way ANOVA analysis of the different parameters considered in this work. The effect of single variable (nutrient availability, NA or magnetic field (MF) intensity) and the variable interaction (NAxMF) was considered. Significant effects (p value < 0.05, 0.001, 0.001) are highlighted in bold

	**p value of two-way ANOVA**		
parameters	**Nutrient availability (NA)**	**Magnetic field (MF) intesity**	**NAxMF**
RL	**<0,001**	**<0.001**	0.3873
SA	**<0.001**	**<0.001**	**<0.05**
Cl	0.2068	0.1766	0.1735
NO3	0.9289	0.4211	0.1935
PO43-	0.5458	0.3222	**<0.05**
SO42-	0.7632	**<0.001**	0.4204
S index	**<0.001**	0.7343	**<0.01**
IRT1	**<0.001**	**<0.05**	**<0.001**
AHA2	0.06537	0.1859	0.6165
FRO2	**<0.001**	**<0.05**	**<0.001**
FIT	**<0.001**	0.1034	0.08463
bHLH38	**<0.001**	**<0.001**	**<0.001**
bHLH39	**<0.001**	0.1238	0.2294
PYE	**<0.001**	**<0.001**	**<0.001**
BTS	0.0002	0.0133	**<0.05**
SULTR1;1	**<0.001**	**<0.001**	**<0.001**
SULTR1;2	0.1604	**<0.001**	**<0.05**
SULTR1;3	**<0.05**	**<0.001**	**<0.01**
SULTR2;1	**<0.01**	**<0.001**	**<0.001**
SULTR2;2	**<0,001**	**<0.001**	**<0.001**
APR1			
APR2			
SPL7	**<0.001**	**<0.001**	**<0.05**
FeSHOOT	**<0.001**	0.4108	0.3649
Fe ROOT	**<0.001**	0.3824	0.2137
Cu shoot	**<0.05**	0.218	0.1345
Cu root	0.5026	**<0.001**	**<0.01**
Mn shoot	0.1322	0.9891	0.4331
Mn root	0.2809	0.2255	0.5634
Zn shoot	**<0.001**	0.9141	0.9805
Zn root	**<0.001**	**<0.05**	0.1783
Mo shoot	**<0.001**	0.3161	0.6244
Mo root	**<0.001**	0.1265	0.3367

Table S2: Macro and micro nutrient composition

Name of the nutrients	Concentration (μM)	Control	-Fe	-S	-Fe-S
$MgSO_4$ $7H_2O$	750	x	x		
MgCl2 6H2O	750			x	x
KH_2PO_4	625	x	x	x	x
NH_4NO_3	1000	x	x	x	x
KNO_3	9400	x	x	x	x
$CaCl_2$ $2H_2O$	1500	x	x	x	x
MES pH5.5 with KOH	1000	x	x	x	x
H_3BO_3	50	x	x	x	x
KI	2.5	x	x	x	x
$ZnCl_2$	15	x	x	x	x
NaFeEDTA	50	x		x	
$CoCl_2$ $6H_2O$	0.055	x	x	x	x
$CuCl_2$ $2H_2O$	0.053	x	x	x	x
$MnCl_2$ $4H_20$	50	x	x	x	x
Na_2 MoO_4 $2H_2O$	0.52	x	x	x	x

Table S3: Primers used in this work

Gene Code	Gene	Forward primer (5'-3')	Reverse primer (5'-3')
**REFERENC GENES**			
At2g37620	*ACT1*	TGCACTTCCACATGCTATCC	GAGCTGGTTTTGGCTGTCTC
At5g19510	*eEF1Balpha2*	ACTTGTACCAGTTGGTTATGGG	CTGGATGTACTCGTTGTTAGGC
At1g13440	*GAPC2*	TCAGGAACCCTGAGGACATC	CGTTGACACCAACAACGAAC
At1g51710	*UBP6*	GAAAGTGGATTACCCGCTG	CTCTAAGTTTCTGGCGAGGAG
**TARGET GENES**			
At4g30190	*AHA2*	AAAGTTGCAGGAGAGGAAGC	GCACGATATCTGAAGCACCA
At1g01580	*FRO2*	TCTCATCAATCCTCGGACCA	TTGTTGGTGTTGTGGTCGAT
At4g19690	*IRT1*	CGGAATAGCGTTAGGGATCG	GCAGCTAGAAGATCCACGAG
At2g28160	*FIT*	GAACATGCTCCTGATGCTCA	ACCCTTTCTCCTCCACTTGT
At3g56970	*BHLH38*	TCAACGGTTTCTGCCACTAG	ACATCCACAAGAACAAACCCA
At3g56980	*BHLH39*	TGTTTCTGTTTCGTCGGAGG	TAATTTTCCTGCGACGGTCA
At5g54680	*ILR3*	GCTGCGAGATGAGAAACAGA	AGTAGGCATCATAGGTGGGG
At5g18830	*SPL7*	GTTGCAGGGATTTTGGGGAG	GGTTCACAGGTTGCTCAGTG
At3g18290	*BTS*	GATTCCCAATGGCAAAGCAC	GATTCCTAGTCCTTCCCCGA

Gene Code	Gene	Forward primer (5’-3’)	Reverse primer (5’-3’)
At3g47640	*PYE*	GACTTGAACACCTCTCCTGC	GGCCTTGGAAAATGGGAAGT
At1g32640	*MYC2*	GTCCGGTTCATTCTCAGACC	CGGAGCTTCGTTTACCTTCA
At1g80830	*NRAMP1*	CGGAACTTATGCTGGACAAT	AGAAGAGGAACCAACGCAAA
At4g04610	*APR1*	CATTGGAGCCAAAAGTTTCGC	TCCTCAATCTCAACCACATCAAC
At1g62180	*APR2*	CGGTGTTGGAAGTCTTGTGA	CTCACACCCGATTGACACAT
At5g01600	*FER1*	TCGTTGAGAGTGAATTTCTGG	ACCCCAACATTGGTCATCTG
At5g23980	*FRO4*	AATAGCGATGTGGGTGACGA	TCATGCAGAACCACGAGTCT
At5g23990	*FRO5*	AGACAAGAAGCCCGAGACAA	GGGTCCAAGAACAGGTGAGA
At5g11260	*HY5*	GGAGTTTGGAGGAGAAGCTG	TTCAGCCGCTTGTTCTCTTT
At4g08620	*SULTR1;1*	TTGCTCAGCCACTTCCGTAC	CTCAAGAGCCTCGAGAAGC
At1g78000	*SULTR1;2*	CCATCCTCGCAGCTATCATC	AGAATGCTCCAATACAGGCG

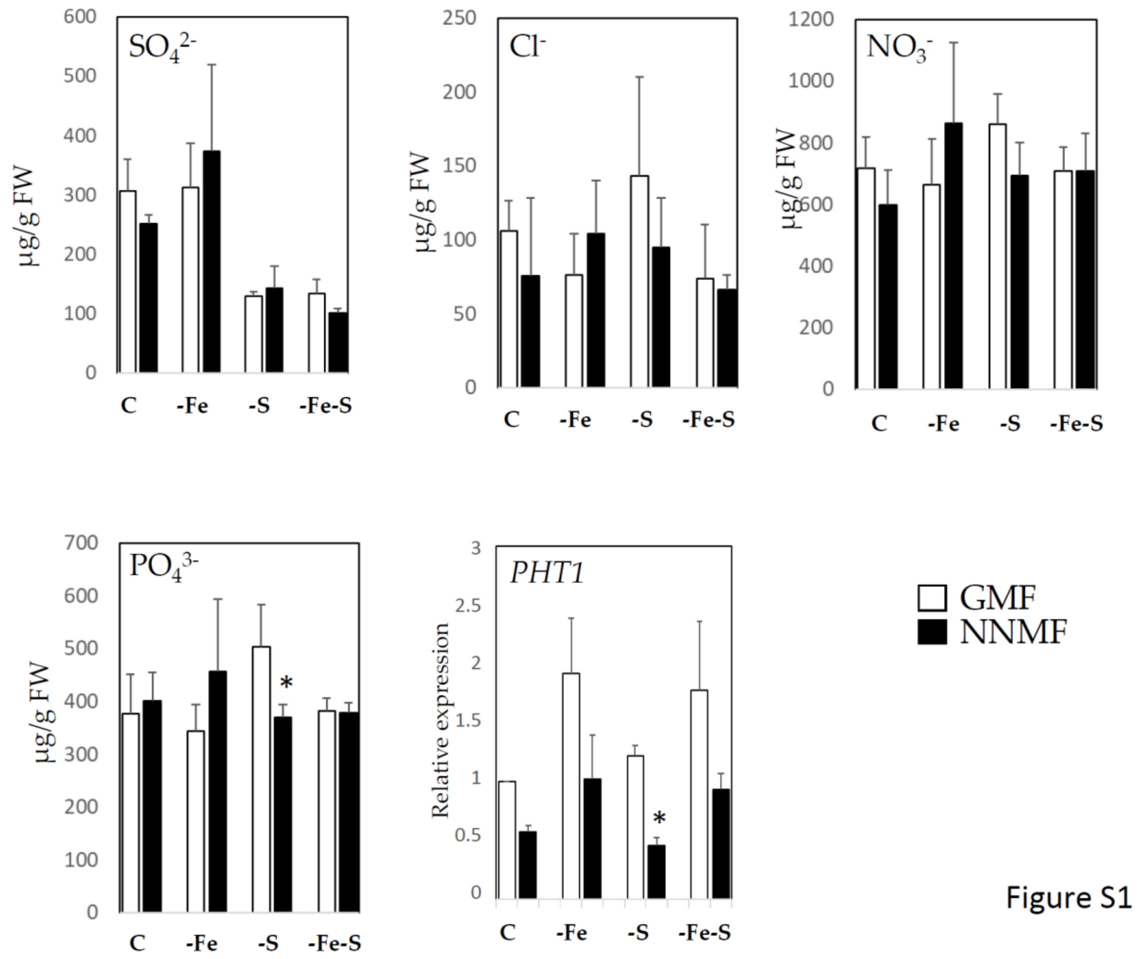

Figure S1. Inorganic anion (upper panel Cl^-, SO_4^{3-}, NO_3^-) concentration and phosphate (PO_4^{3-}) and PHT1 gene expression (lower panel) of Arabidopsis thaliana seedlings and grown under full nutrient condition (C), absence of iron (-Fe), absence of sulfur (-S) and combined Fe and S deficiency (-Fe-S). Determination were performed 7 days after transferring seedlings to NNMF conditions. Mean value (±SE) are from 3 independent biological experiments and asterisk indicate statistical difference ($p<0.05$) between GMF- and NNMF-exposed plants. Two-way ANOVA results are reported in the Table S1

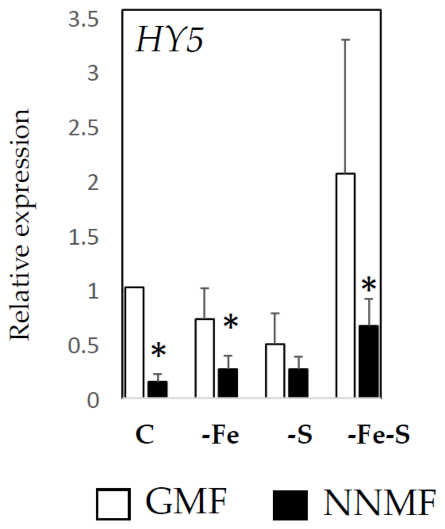

Figure S2. Expression of gene HY5 in Arabidopsis thaliana seedlings grown under full nutrient condition (C), absence of iron (-Fe), absence of sulfur (-S) and combined Fe and S deficiency (-Fe-S). Plants were exposed for 7 days both to GMF and NNMF conditions. Data are from three independent experiment (n=3) and asterisk indicate statistical difference ($p<0.05$) between GMF- and NNMF-exposed plants. Values are expressed as fold change (SE) with respect to control plants growing in GMF conditions under full nutrient media (C). Two-way ANOVA results are reported in the Table S1
